# Assessment of potential myopia risk factors, including chronotype, in Estonian adolescents: a cross-sectional study

**DOI:** 10.1186/s12886-024-03747-5

**Published:** 2024-11-05

**Authors:** Teele Palumaa, Delis Linntam, Reili Rebane, Kristel Harak, Mari Tamsalu, Katrin Sõnajalg, Karina Ülper, Svetlana Belova, Triin Keller, Marika Tammaru, Kadi Palumaa

**Affiliations:** 1 Eye Clinic, East Tallinn Central Hospital, Ravi 18, Tallinn, 10138 Estonia; 2 Institute of Genomics, University of Tartu, Tartu, Estonia; 3 Department of Ophthalmology, Emory University, Atlanta, USA; 4 Research Department, East Tallinn Central Hospital, Tallinn, Estonia

**Keywords:** Myopia, Risk factors, Chronotype, Circadian rhythms

## Abstract

**Background:**

Myopia is a growing healthcare concern worldwide. Increasing evidence suggests that sleep and circadian rhythms may be associated with myopia. Furthermore, the risk factors of myopia have not been studied in the Estonian population to date. This study aimed to evaluate chronotype, lifestyle factors, and parental myopia in relation to myopia in Estonian secondary school students.

**Methods:**

Grade 10 students from three secondary schools in Tallinn, each with distinct focuses: one science-oriented, one arts-oriented, and one sports-oriented, were invited to participate. They underwent a comprehensive ocular examination, including cycloplegic autorefraction and ocular biometry. Chronotype was evaluated with the Morningness − Eveningness Questionnaire. Participants reported parental myopia and replied to a set of questions, separately for schooldays and free days, to indicate the amount of time they spent outdoors, doing near work and intermediate distance activities. Myopia was defined as cycloplegic SER ≤ − 0.50 D. Logistic regression analysis was performed to assess the association of the studied factors with myopia.

**Results:**

A total of 123 students (57% female) participated in the study, with a mean age of 16.71 years (standard deviation 0.41). In a multivariable regression model, having two myopic parents was associated with higher odds of myopia (OR 3.78, 95% CI 1.15 − 12.42). We found no association between myopia and chronotype. Notably, time spent outdoors and doing near work or intermediate distance work did not affect the likelihood of having myopia. We observed that students attending the sports-oriented school had lower odds of myopia than those attending the science-oriented school (OR 0.12, 95% CI 0.03–0.51).

**Conclusion:**

Chronotype was not associated with myopia in our study sample. Consistent with previous reports, we identified parental myopia as a myopia risk factor. Interestingly, there was no association between myopia and time spent outdoors or near work. However, the odds of myopia varied depending on the school attended by the participants, which may reflect the educational load or lifestyle of participants in earlier childhood.

## Background

The prevalence of myopia, or short-sightedness, has increased markedly over the last decades, particularly in East Asia, where myopia was estimated to affect 52% of the population in 2020 [1]. For example, in South Korea, myopia prevalence in 19-year-old males in Seoul was estimated at 96.5% in 2010 [2]. Should the current trend continue, approximately half the global population will be myopic by 2050 [1]. While the prevalence of myopia is lower in Europe than in East Asian countries, it has similarly been increasing over the recent decades, with an estimated prevalence of 32–37% in 2020 [2].

The prevalence of myopia has not been studied in Estonia. However, neighbouring countries like Sweden report a low myopia prevalence in schoolchildren, with only 10% affected [3]. A similar low prevalence has been observed elsewhere in Scandinavia, with 13% in Norwegian adolescents [4]. Myopia prevalence has been linked to educational performance, with countries scoring higher in the Programme for International Student Assessment (PISA) showing increased rates of myopia [5]. PISA evaluates the knowledge and skills of 15-year-old students in mathematics, sciences, and functional reading [6]. Estonian students have performed very well throughout the years. Among the 81 countries that participated in 2022, Estonian students placed 1st in Europe in natural sciences and shared 1st -2nd place in Europe in mathematics and functional readings. Globally, they ranked 6th in science and reading and 7th in mathematics, following East Asian countries [6]. Based on these data, it is challenging to estimate myopia prevalence in Estonia. Although nearby countries report low myopia rates, Estonia’s high educational performance, compared to other European countries, may suggest higher myopia rates.

Myopia risk factors have been studied extensively worldwide, yet such studies are lacking in the Estonian population. It has emerged that the risk of developing myopia is reduced in children who spend more time outdoors [7–10] and increased with more near-work [11], and computer use [12]. Several recent studies have raised the possibility that myopia is associated with circadian rhythms. For example, genes associated with refractive errors in genome-wide studies have an increased representation of genes controlling circadian rhythms [13]. It is also known that axial length and choroidal thickness show diurnal rhythmicity in humans [14, 15] and chicks [16, 17]. There are several lines of evidence suggesting that myopia can alter ocular diurnal rhythms. For example, one study demonstrated that inducing myopia in chicks with form deprivation abolished this circadian rhythmicity in axial length [16]. Another study found that while the circadian rhythm of axial length was preserved in animals developing myopia, the timing of peak axial length changed [18].

There is also evidence to suggest that myopia may be linked to sleep. For example, myopic children have been reported to sleep later than children with no myopia [19–22], and to have worse sleep quality [19, 23]. However, the data in the literature is inconsistent and several studies have found no associations between myopia and sleep quality [24] and bedtime [25–28]. Chronotype, or one’s sleep-wake timing preference, has been studied in the context of myopia in a couple of studies, and no definitive associations have been found. Two studies have reported that myopia is associated with late chronotype [29, 30]. Yet, in one report, the circadian phases of myopes and non-myopes did not differ [31] and in another, self-report of a “morning” or “evening” type was not associated with myopia progression [21]. In conclusion, the current literature contains conflicting results on the associations between myopia and circadian parameters, and therefore, further studies are needed.

There are several mechanisms, which may underlie the potential link between circadian rhythms, sleep and myopia. For example, it has been demonstrated that individuals with a later chronotype spend less time in daylight [32, 33], which, in turn, is a well-characterised myopia risk factor [7–10]. Daylight is significantly brighter than indoor light levels, and bright light exposure results in dopamine release in the retina [34, 35], which has been shown to inhibit eye growth in several animal studies [36, 37]. Furthermore, dopamine, in concert with melatonin, is a crucial neurotransmitter setting the circadian rhythms of the retina [38]. It may be the downstream effect of aberrant retinal circadian rhythm regulation that leads to myopic eye growth. The association between sleep quality and myopia may also be associated with an effect of chronotype, as evening chronotype has been associated with poor sleep quality [39–41].

In conclusion, there are several possibilities whereby circadian rhythms and sleep may be associated with myopia, yet the results from studies conducted thus far are inconsistent. Therefore, we aimed to assess the effect of chronotype, in addition to parental myopia status, and lifestyle factors, on myopia in Estonian adolescents.

## Methods

The study was conducted at the East Tallinn Central Hospital Eye Clinic in 2019. It adhered to the principles of the Declaration of Helsinki and was approved by the Tallinn Medical Research Ethics Committee. Grade 10 students from three public schools in Tallinn were invited to participate, and a total of 123 students were recruited. Written informed consent was obtained from the participants and their parents or legal guardians. All participants were European Caucasians. The schools differed slightly in their education profile, putting more emphasis on science, arts, or sports.

In Estonia, the education system begins with primary school at 7 years of age and is divided into basic education (grades 1–9) and secondary education (grades 10–12). Before basic school, children have the opportunity to attend preschool. Students typically start grade 10, the first year of secondary education, at age 16. Many transfer schools at this stage to better align with their interests. In Estonia, schooling is mandatory until the completion of basic education or the age of 17 [42]. 

In this study, participants underwent a comprehensive ocular examination, including cycloplegic refraction, ocular biometry and biomicroscopy. Cycloplegia was achieved by applying 1% cyclopentolate (1 drop) and 0.5% tropicamide (1 drop) twice with a 5-minute interval, as per [8]. Cycloplegia was evaluated 40 min after the application of first eye drops and was considered adequate when the pupil was ≥ 6 mm in diameter and not responsive to light. Ocular refraction was measured with the hand-held HandyRef-K autorefractor (Nidek, Tokyo, Japan). The HandyRef-K autorefractor demonstrates an accuracy within ± 0.25 dioptres (D) for spherical and cylindrical vertex power measurements [43], which is in accordance with the International Organization for Standardization (ISO) standards. Its spherical equivalent readings are in high agreement with other autorefractors, including those of the table-mounted Topcon TRK-2P autorefractor, showing an average difference of 0.11 D toward more negative values with the HandyRef-K, which is within clinically acceptable limits [44]. Axial length and anterior corneal curvature were measured with IOLMaster 500 (Zeiss, Oberkochen, Germany), which has very good agreement in biometry measurements with newer devices, such as IOLMaster 700 [45]. SER was calculated as sphere + ½ cylinder, and participants’ SER was defined as the mean SER of the left and right eyes. Myopia was defined as cycloplegic SER ≤–0.50 D, high myopia as SER ≤–6.00 D, astigmatism as ≤–1.00 dioptre cylinder and hyperopia as SER ≥ 2.00 D.

A set of questions were administered to the participants to analyse their daily activities. The activities defined as near work and intermediate distance work were defined based on the study by Rose et al. [8]. In their study, near work activities included drawing, homework, reading, and handheld computer use [8]. Intermediate distance activities were a sum of watching television, playing video games and using the computer [8]. The English translations of the questions used in this study are: (1) How many hours do you spend outdoors? (2) How many hours do you spend doing near work (for example, reading, writing, using a mobile phone or a tablet)? (3) How many hours do you spend using the computer? (4) How many hours do you spend watching the TV and/or playing video games? Questions 3 and 4 were summed to get the value for intermediate viewing distance activities. These four questions were asked separately for school days and free days. The total time per week was calculated and used in the analysis. While this set of questions has not undergone formal validation in the context of comparing self-reported data to objective measures, we took care to ensure that the questions were clear and comprehensively captured the key activities previously defined in studies on near work and intermediate distance work. Furthermore, while self-reporting is known to introduce both random and non-random errors, we do not have reason to believe that these errors would systematically differ between myopes and non-myopes, thus minimising the risk of introducing systematic reporting bias.

Chronotype was evaluated with the Morningness-Eveningness Questionnaire (MEQ) [46]. MEQ was translated from English into Estonian and pilot-tested on a separate cohort of thirty 16-17-year-olds to eliminate any misunderstandings in the wording. The participants also replied to a question about parental myopia.

Statistical analyses were performed using Stata Software for Statistics and Data Science (version 14.2). Graphs were produced in Prism (version 9, GraphPad). Descriptive variables are reported as number and proportion, continuous variables with a normal distribution are presented as mean and standard deviation (SD). For comparisons between groups, two-sided t-tests were used for variables with a normal distribution. Pearson’s Chi-squared test was used to evaluate the association between categorical demographic characteristics and myopia status, assessing whether the distribution of these characteristics differed significantly between myopes and non-myopes. Variables investigated in the context of myopia were assessed with multivariable logistic regression analysis, with myopia status as the dependent variable; and gender, parental myopia, school, chronotype, outdoor time, near work and intermediate distance activity time as independent variables. The results were presented as odds ratios (OR) with their 95% confidence interval (CI). Spearman rho was calculated to characterise correlations between ocular parameters. A *p* value of < 0.05 was considered statistically significant.

## Results

A total of 123 students participated in the study (57% females), the mean age (± SD) was 16.71 ± 0.41 years, age range 15.2–17.7 years). Half of the participants were from the science-oriented school, 28% from the arts-oriented and 22% from the sports-oriented school. 18% reported having two myopic parents, and 41% had one myopic parent (see Table 1 for a demographic characterisation). The prevalence of myopia in our study population was 30.9%, high myopia was present in 2.4%, hyperopia in 3.3% and astigmatism in 11.4%.

**Table 1:** Characteristics of the study population

Characteristic	All participants	Non-myopes	Myopes	*p* value
	*N* = 123 (100%)	*N* = 85 (69%)	*N* = 38 (31%)	
**Sex**,** n (%)**				0.35^1^
Female	53 (43%)	39 (46%)	14 (37%)	
Male	70 (57%)	46 (54%)	24 (63%)	
**Age**,** mean (SD)**	16.71 (0.41)	16.68 (0.43)	16.76 (0.37)	0.32^2^
**Parental myopia**,** n (%)**				0.032^1^
None	43 (35%)	35 (41%)	8 (21%)	
One myopic parent	58 (47%)	39 (46%)	19 (50%)	
Both parents myopic	22 (18%)	11 (13%)	11 (29%)	
**School**,** n (%)**				0.004^1^
Science	61 (50%)	34 (40%)	27 (71%)	
Arts	35 (28%)	27 (32%)	8 (21%)	
Sports	27 (22%)	24 (28%)	3 (7.9%)	
**Chronotype (MEQ score)**,** n (%)**				0.54^1^
I tercile (27–43, evening types)	41 (33%)	31 (36%)	10 (26%)	
II tercile (44–50)	42 (34%)	28 (33%)	14 (37%)	
III tercile (51–72, morning types)	40 (33%)	26 (31%)	14 (37%)	
**Time spent outdoors (hours per week)**,** n (%)**				0.96^1^
I tercile (4.5–11.0)	40 (33%)	27 (32%)	13 (34%)	
II tercile (11.25–17.75)	33 (27%)	23 (27%)	10 (26%)	
III tercile (18.0–43.5)	50 (41%)	35 (41%)	15 (39%)	
**Time spent on near work (hours per week)**,** n (%)**				0.37^1^
I tercile (14.0–45.0)	39 (32%)	28 (33%)	11 (29%)	
II tercile (46.0–59.0)	43 (35%)	32 (38%)	11 (29%)	
III tercile (60.0–95.0)	41 (33%)	25 (29%)	16 (42%)	
**Time spent on intermediate distance work (hours per week)**,** n (%)**				0.32^1^
I tercile (3.0–15.0)	41 (33%)	30 (35%)	11 (29%)	
II tercile (15.5–24.5)	40 (33%)	24 (28%)	16 (42%)	
III tercile (25.0–68.0)	42 (34%)	31 (36%)	11 (29%)	

The number and proportion of myopes and non-myopes in each demographic group are presented. The Pearson’s Chi-squared test (^1^) was used to assess the differences in characteristics between myopes and non-myopes. Two-tailed t-test (^2^) was used to analyse the difference in age between myopes and non-myopes

**Fig. 1:**
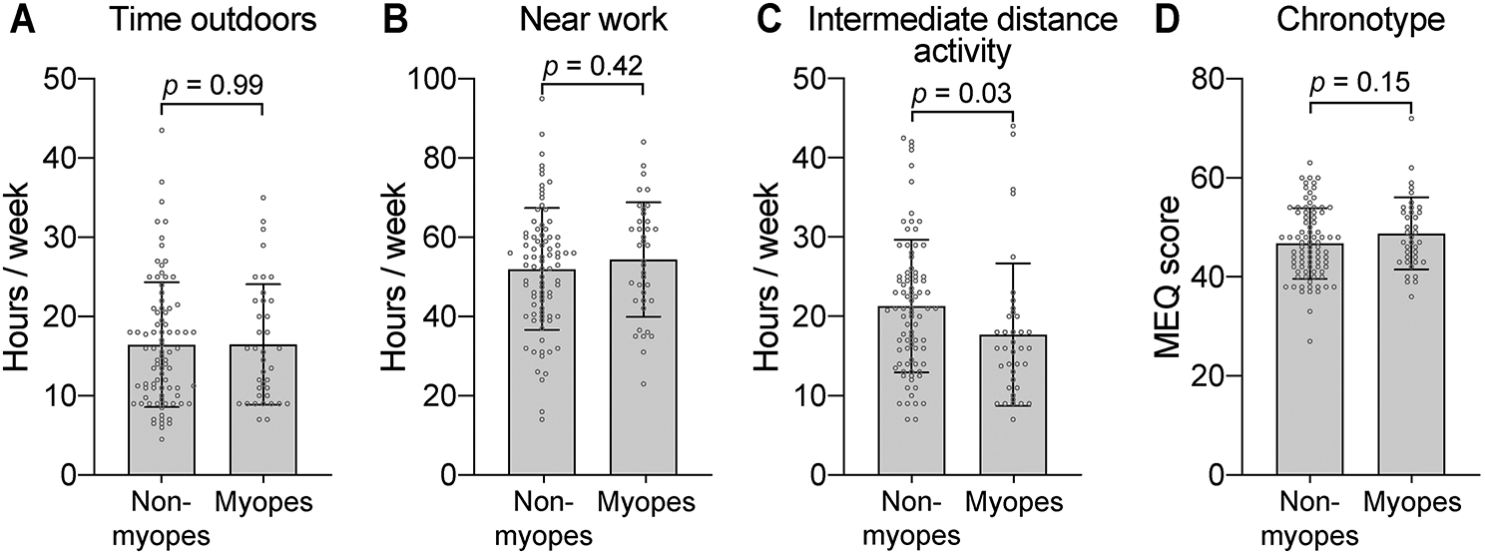
Distribution of factors analysed in non-myopes and myopes. (**A**) Time spent outdoors, (**B**) on near work and (**C**) intermediate distance activities in non-myopes and myopes. (**D**) Distribution of chronotype, measured with MEQ, in non-myopes and myopes. Data are presented as mean ± SD. Statistical analyses were performed using two-tailed t-tests. MEQ, Morningness-Eveningness Questionnaire; SD, standard deviation

The average time spent outdoors per week was 16.5 h for both non-myopes and myopes (Fig. 1A). Myopes spent slightly more time on near-work activities, 54.4 h, compared to 52.0 h per week in non-myopes, however, the difference was not statistically significant (*p* > 0.05, two-tailed t-test, Fig. 1B). Interestingly, non-myopes spent on average more time on intermediate distance activities (21.3 h) compared with non-myopes (17.7 h) (*p* = 0.03, Fig. 1C). In MEQ, a larger score indicates an earlier chronotype [46]. In our study sample, there was no statistically significant difference in MEQ score between the two groups: the average MEQ score was 46.7 for non-myopes and 48.8 for myopes (*p* > 0.05, Fig. 1D).

Multivariable logistic regression analyses of the factors studied revealed that participants with two myopic parents were 4.3 times more likely to have myopia (95% CI 1.19–15.74) (Table 2). Subjects attending the sports-oriented school had lower odds of myopia than participants attending the science-oriented school (OR 0.12, 95% CI 0.03–0.51). We found no significant association between chronotype and refractive status. Time spent outdoors, on near work and intermediate distance activities were not associated with myopia in our study.

**Table 2:** Effect of demographic and lifestyle factors and chronotype on myopia status

Characteristic	Odds ratio	*p* value	95% CI
**Sex**			
Female	1.00		
Male	1.83	0.213	0.71–4.76
**Parental myopia**			
None	1.00		
One myopic parent	1.50	0.459	0.52–4.33
Both parents myopic	4.32	**0.026**	1.19–15.74
**School**			
Science	1.00		
Arts	0.41	0.103	0.14–1.20
Sports	0.12	**0.004**	0.03–0.51
**Chronotype (MEQ score)**			
I tercile (27–43, evening types)	1.00		
II tercile (44–50)	2.29	0.158	0.73–7.26
III tercile (51–72, morning types)	2.69	0.074	0.91–7.98
**Time spent outdoors (hours per week)**			
I tercile (4.5–11.0)	1.00		
II tercile (11.25–17.75)	1.17	0.794	0.35–3.90
III tercile (18.0–43.5)	1.00	0.996	0.32–3.14
**Time spent on near work (hours per week)**			
I tercile (14.0–45.0)	1.00		
II tercile (46.0–59.0)	0.58	0.363	0.18–1.86
III tercile (60.0–95.0)	1.00	0.996	0.32–3.15
**Time spent on intermediate distance work (hours per week)**			
I tercile (3.0–15.0)	1.00		
II tercile (15.5–24.5)	1.88	0.270	0.61–5.76
III tercile (25.0–68.0)	0.97	0.961	0.30–3.19

A multivariable logistic regression model was fitted with myopia status as the dependent variable. Sex, parental myopia, school, chronotype tercile, outdoor time tercile, near work tercile and intermediate distance activity time tercile were included as independent variables. A *p* value of < 0.05 was considered statistically significant and marked in bold. CI, confidence interval; MEQ, Morningness-Eveningness Questionnaire

**Fig. 2:**
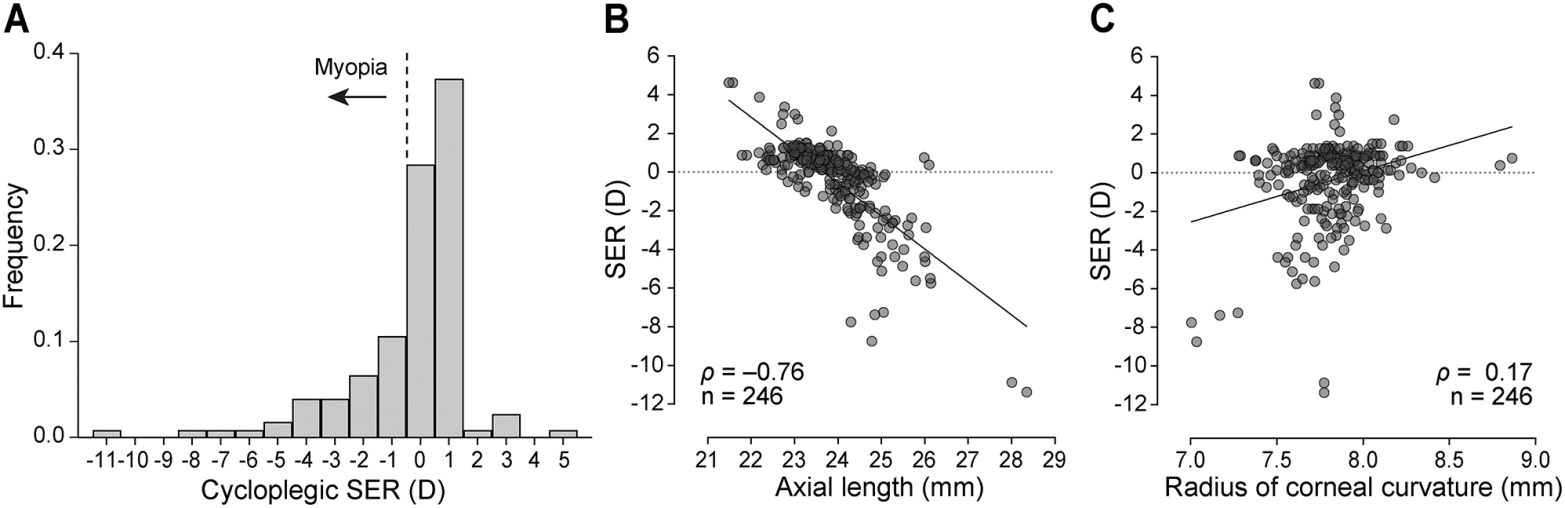
Characterisation of SER of the study population. (**A**) Distribution of the participants’ mean cycloplegic SER grouped into one dioptre bins. Correlations between (**B**) SER and axial length, and (**C**) SER and radius of corneal curvature in individual eyes (Spearman correlation, individual values fitted with simple linear regression to illustrate the relationship). SER, spherical equivalent refraction; D, dioptres

The SER values displayed a left-skewed distribution in our study population with the median cycloplegic SER + 0.25 D and mean SER − 0.38 D (Fig. 2A). Myopia exhibited both an axial and corneal component as more myopic refraction was correlated with both longer axial length (*r* = − 0.76, Fig. 2B), and smaller radius of corneal curvature, or steeper corneas (*r* = 0.17, Fig. 2C).

## Discussion

This is the first study to investigate myopia risk factors in Estonia. Chronotype, assessed with MEQ, was not associated with myopia in our sample. Although later bedtime has been associated with myopia in several studies [19–22], the link between chronotype and myopia has been inconsistent across studies. In their investigation, Flanagan et al. [31] found that myopes had higher melatonin levels at all time points studied, while the circadian parameters, i.e. dim-light melatonin onset (DLMO), phase of circadian activity determined with actigraphy, and MEQ score did not differ between myopes and non-myopes. Furthermore, self-reported “morning” or “evening” type was also not associated with myopia progression in a study by Lee et al. [21]. On the other hand, Chakraborty et al. [30, 47] found that myopic adolescents [47] and children [30] have a later chronotype than age-matched emmetropes, quantified as a delay in their DLMO as well as a lower MEQ score. The lack of detectable association in our study may be due to a limited sample size, reducing our ability to capture subtle effects. Additionally, while the MEQ is in good agreement with more objective measures of circadian timing, such as DLMO [48] and actigraphy [49], it remains a subjective assessment, and the use of more objective tools, may provide a more accurate chronotype profile. The studies by Chakraborty et al. [30, 47] were conducted in Australian populations, and it is also possible that population differences could influence the relationship between chronotype and myopia. Future research with larger sample sizes and objective chronotype measurements would help further understand the role of circadian rhythms in myopia. In addition, it would be beneficial to track changes in chronotype and myopia progression over time in longitudinal studies to understand the causality of the potential relationship. The field would also benefit from genetic analyses to elucidate the genetic predisposition to both circadian rhythm variations and myopia, which would help identify shared genetic pathways or regulatory mechanisms that could influence refractive development and circadian regulation.

In agreement with other investigations [50], we determined parental myopia as a myopia risk factor in the Estonian population. While increased time spent outdoors has been convincingly shown to protect from myopia development in multiple studies [7–10], we did not see such a correlation in our study population. The reason behind this observation may be our limited sample size and assessing time spent outdoors with a single question. A more detailed measure of daily activities would have enabled a more accurate assessment of time spent in daylight. It has been demonstrated that a daily activities diary correlates better with time spent outdoors evaluated by wearable light meters than a questionnaire [51]. However, this is not the first study to report no association between time outdoors and myopia in adolescents. Schmid et al. [52] found no differences in the time spent in sunlight between 17 to 25-year-old myopes and non-myopes. Lu et al. [53] reported no differences in self-reported time spent outdoors and myopia status in Singaporean adolescents (mean age 14.7 years). Furthermore, Hagen et al. [4] saw no differences in reported outdoor time between myopes and non-myopes in 16 to 19-year-old Norwegian adolescents. Our study participants were aged between 15 and 17 years, and it may be that outdoor time at a younger age, which is more critical for refractive development, confers a protective effect, and outdoor time during adolescence may not be reflective of the previous outdoor exposure.

Near work was also not associated with myopia in our study. The relationship between near work and myopia has been reported in some [54], but not other studies [3, 55]. Furthermore, the beneficial effect of outdoor time outweighs the detrimental effect of increased near work on myopia [8, 55]. Nevertheless, meta-analyses suggest that while the effect is not large, increased near work is associated with higher odds of myopia [11, 56]. In this study, we assessed the total amount of time spent on near work activities with a single question, which does not capture the nature of the activities. It could be that the intensity of near work, for example, the reading distance and continuous reading, not the total amount of time on near work, is associated with myopia [54]. Future studies could use more detailed questionnaires to assess time spent on near work and near work intensity to further analyse the impact of near work on myopia in the Estonian population.

In our study population, the prevalence of myopia was 31%. While it was not a prevalence study, all grade 10 students from the three schools included were invited to participate. Based on published myopia prevalence reports, it has been estimated that the myopia prevalence in 15-year-olds in Europe is approximately 35% [57], which aligns well with our findings. However, a prevalence of 31% would be significantly higher than the reported prevalence in countries in proximity to Estonia, such as the Scandinavian countries Sweden and Norway, where the figures are 10% and 13%, respectively [3, 4]. Given that Estonian adolescents rank in the top of European countries in academic performance [6], myopia prevalence would be expected to be higher than on average in Europe. However, a prevalence study is needed to estimate myopia prevalence in Estonia.

Interestingly, we found that individuals attending the school that focused more on sports had lower odds of myopia compared to those attending the school that focused on sciences. The grade sampled was the first grade of secondary school, a stage when many students change schools based on their interests. Students attending the science-oriented school may have been exposed to an educational environment with higher educational loads, such as more homework, which has been demonstrated as a factor associated with myopia [58]. A higher educational load could have resulted in more near work and less outdoor activity. In contrast, those attending the sports-oriented school had likely engaged more in physical activities throughout their lives, reducing the time available for near work. Additionally, sports activities often occur outdoors, increasing exposure to daylight. We hypothesise that the observed school-related effect on myopia reflects a combination of past lifestyle factors, such as educational load, time spent outdoors and amount of near work activities. These differences in daily habits likely contribute to the lower rates of myopia among students attending the sports-oriented school compared to those in the science-oriented school.

## Conclusions

In conclusion, this is the first study evaluating myopia risk factors in Estonia. In agreement with published work, we found that parental myopia was a risk factor for myopia. Decreased time spent outdoors and increased time on near-work activities were not associated with myopia in our study sample. In addition, we investigated whether chronotype affected myopia development and found that in our study sample, chronotype, assessed with MEQ, was not associated with myopia. Interestingly, we found that individuals attending a sports-oriented school were less likely to have myopia compared to those attending a science-oriented school.

## Data Availability

All data generated or analysed during this study are included in this published article.

## Abbreviations

CI: Confidence interval
D: Dioptre
MEQ: Morningness–Eveningness Questionnaire
OR: Odds ratio
SD: Standard deviation
SER: Spherical equivalent refraction
